# Challenges Associated With Re‐Emergence of Lassa Fever in Nigeria: An Exploratory Study of Epidemiology, Phylogenomics, and Recommendations Toward Its Eradication

**DOI:** 10.1002/hsr2.70225

**Published:** 2024-11-29

**Authors:** Ridwan Olamilekan Adesola, Ibrahim Idris, Adetolase Azizat Bakre, Joseph Fosu Arthur, Joanna Nicole D'Souza

**Affiliations:** ^1^ Department of Veterinary Medicine, Faculty of Veterinary Medicine University of Ibadan Ibadan Nigeria; ^2^ Department of Veterinary Medicine, Faculty of Veterinary Medicine Usmanu Danfodiyo University Sokoto Nigeria; ^3^ Department of Clinical Microbiology, College of Health Sciences Kwame Nkrumah University of Science and Technology (KNUST) Kumasi Ghana; ^4^ Department of Biotechnology R.V. College of Engineering Bangalore India

**Keywords:** Africa, Lassa fever, Nigeria, One Health, phylogenomics

## Abstract

**Background:**

Lassa fever (LF) is a viral hemorrhagic illness endemic in Nigeria and other West African countries. In recent years, the number of reported cases of LF in Nigeria has increased. In this study, we discussed the epidemiology of LF, the phylogenomics of the LF virus, issues associated with the increased cases of LF, and recommendations for tackling the future occurrence of LF in Nigeria.

**Methods:**

Epidemiology data on LF were obtained from the Nigeria Centre for Disease Control database and analyzed using Microsoft Excel software. About 59 partial and complete sequences consisting of both small and large segments of the LF virus were retrieved from the National Center for Biotechnology Information from 1969 to 2013 to study the evolutionary relationship of the LF virus in Nigeria.

**Results:**

Nigeria has been shown to have the highest prevalence of Lassa fever among African countries, with seasonal occurrence in both wet and dry seasons. Furthermore, the phylogenetic analysis of the LF virus showed a great relationship with several outbreaks of LF in Nigeria and other African countries.

**Conclusion:**

To combat the increasing cases of LF in Nigeria, there is a need to increase the molecular diagnosis capacity in Nigeria, improve public health awareness about the disease in rural and urban settlements, integrate a surveillance system through the One Health lens, and support LF vaccine research in Africa.

## 1 Introduction

Lassa fever (LF) is a zoonotic acute viral hemorrhagic disease. The World Health Organization and the Centers for Disease Control and Prevention estimate that there are 5000 fatal cases of LF per year in West Africa, with an annual case report between 100,000 and 300,000 [1]. LF virus was initially discovered in Lassa town, Borno state, Nigeria, in 1969 [2]. Since then, Nigeria has been battling with different outbreaks. Nigeria experienced its highest annual incidences of LF between 2018 and 2023 (633 confirmed cases in 2018, 833 in 2019, 1189 in 2020, 510 in 2021, 1067 in 2022, 1270 in 2023, across 29 states, and 125 Local Government Areas) [3]. This has led to the mobilization of national and international healthcare resources and raised concerns about the ongoing, systematic emergence of LF in Nigeria.

LF is caused by Lassa fever virus (LFV), a single‐stranded RNA virus belonging to the *Arenaviridae* family [4]. The LFV' genome comprises two segments, the L (7.3 kb) and S (3.4 kb), each of which codes for two proteins. The small (S) segment encodes the precursors of nucleoproteins and glycoproteins, whereas the large (L) segment encodes zinc‐binding proteins and viral RNA polymerase [3]. LFV is categorized into four lineages: I, II, III, and IV [5]. Lineages I, II, and III are endemic to Nigeria. Meanwhile, lineage IV is endemic to Guinea, Sierra Leone, Côte d'Ivoire, Mali, and Liberia [5]. Recently, a study has found the occurrence of new lineages, V and VI, in Côte d'Ivoire, Mali, and Nigeria [6]. Nonetheless, reports of different LFV lineages have been found throughout West Africa.

Multimammate rats (*Mastomys natalensis*) are the LFV's natural host and primary disease vectors. They are presumably the most prevalent rodent in tropical Africa, primarily found in rural regions and more frequently within homes than outdoors [7]. Members of the genus are carriers of the virus. Humans are infected with LF through physical contact or ingestion of multimammate rats. In some regions, up to 90% of people eat rodents, considered a delicacy [8]. Person‐to‐person transmission can also occur, especially among laboratory and hospital workers [9]. Serological studies show that the virus commonly spreads in hospital and community settings [10]. In endemic nations, nosocomial transmission is facilitated by inadequate prevention and control measures in healthcare facilities [11]. LFV can further be transmitted via sexual intercourse due to the ability of the virus to infect sperm cells [12].

The symptoms of LF emerge 2–21 days after infection [13]; symptoms include diarrhea, hemorrhage, confusion, respiratory distress, stomach discomfort, facial edema, hearing loss, encephalitis, and tremor [13, 14]. Clinical laboratory findings of LF are thrombocytopenia, leucopenia, increased blood, proteinuria, and increased liver enzyme concentration [15, 16]. Due to the lack of specificity in the clinical signs and symptoms of LF, which can lead to a wide range of differential diagnoses, especially in the early phase of the disease, laboratory diagnosis is crucial for timely and effective treatment [17]. Accurate laboratory diagnosis is essential for triage, the deployment of barrier nursing, contact tracing, and the beginning of ribavirin treatment, especially in acute cases. For case confirmation, reverse transcription‐polymerase chain reaction is frequently utilized [18]. The gold standard for diagnosing the LFV is virus isolation; however, because BSL‐4 biocontainment is necessary, it is not feasible in locations where the virus is endemic [17]. Effective diagnosis of LF can only be achieved following laboratory confirmation, a significant challenge in developing countries like Nigeria. Other diagnostic methods apart from virus isolation are serology and molecular techniques. Patients with LF have been successfully treated with the antiviral medication ribavirin. It has been demonstrated that early in the course of the sickness is when it is most effective. Supportive care for patients should also include the management of any other aggravating infections, as well as the preservation of optimal fluid and electrolyte balance, oxygenation, and blood pressure [19].

Few studies have worked on the molecular epidemiology of LFV to study the intrinsic challenges associated with the re‐emergence of LF in Nigeria through genetic diversity [5, 20]. Through a molecular epidemiological approach, the evolution and spread of the LF virus can be tracked in real‐time and help with routine LF diagnosis. This paper aims to discuss the epidemiology of LF, the phylogenomics of the LF virus, problems associated with the increased cases of LF, and recommendations for tackling the future occurrence of LF in Nigeria.

## 2 Materials and Methods

### 2.1 LF in Nigeria

Epidemiological data about the LF outbreaks in Nigeria were retrieved from Nigeria Centre for Disease Control (NCDC) database (https://ncdc.gov.ng/diseases/sitreps/?cat=5&name=An%20update%20of%20Lassa%20fever%20outbreak%20in%20Nigeria) [3]. Microsoft Excel version 365 was used to sort and analyze data.

### 2.2 Phylogenetic Analysis

We retrieved 59 partial and complete coding sequences consisting of both S and L segments of the LFV from the National Center for Biotechnology Information (NCBI) website (https://www.ncbi.nlm.nih.gov/nuccore) from 1969 to 2013 (Table 1). Twenty (L) and twenty‐five (S) sequences were downloaded from Nigeria, while eight (L) and six (6) were from other African countries. All the retrieved sequences were aligned using Bioedit software version 7.2. The evolutionary relatedness of the virus was conducted in MEGA 11 [21]. The phylogenetic tree was constructed using the Maximum Likelihood method based on the Tamura 3‐parameter model and selecting the topology with a superior log likelihood value [22]. The tree length in which the associated taxa clustered is shown next to the branches. The tree was drawn to a scale of 0.50.

**Table 1:** Metadata of Lassa fever virus sequences isolated in Nigeria from 1969 to 2013.

Segments	Year of isolation	Accession number	Hosts	Isolation source	Location
**Large**	1969	KM822127	Not provided	Not provided	Nigeria
	2004	AY693639	Not provided	Not provided	Nigeria
	2004	AY693640	Not provided	Not provided	Nigeria
	2005	GU481059	Humans	Serum	Ebonyi State
	2005	GU481057	Humans	Serum	Ebonyi State
	2008	GU481069	Humans	Serum	Ebonyi State
	2008	GU481071	Humans	Serum	Plateau State
	2008	GU481073	Humans	Serum	Plateau State
	2008	GU481065	Humans	Serum	Federal Capital Territory
	2008	GU481062	Humans	Serum	Plateau State
	2008	GU481067	Humans	Serum	Ebonyi State
	2009	KM822108	Humans	Not provided	Nigeria
	2009	KM822100	Humans	Not provided	Nigeria
	2009	KM822104	Humans	Not provided	Nigeria
	2009	KM822096	Humans	Not provided	Nigeria
	2009	KM822106	Humans	Not provided	Nigeria
	2009	KM822102	Humans	Not provided	Nigeria
	2009	KM822098	Humans	Not provided	Nigeria
	2009	KM822094	Humans	Not provided	Nigeria
**Small**	1969	KM822128	Not provided	Not provided	Nigeria
	2004	DQ010030	Humans	Serum	Edo State
	2004	DQ010031	Humans	Not provided	Nigeria
	2005	GU481058	Humans	Serum	Ebonyi State
	2007	GU481060	Humans	Serum	Plateau State
	2007	GU481061	Humans	Serum	Plateau State
	2008	JQ511991	Humans	Serum	Edo State
	2008	JQ511992	Humans	Serum	Edo State
	2008	GU481063	Humans	Serum	Federal Capital Territory
	2008	GU481064	Humans	Serum	Federal Capital Territory
	2008	GU481066	Humans	Serum	Ebonyi State
	2009	KM822103	Humans	Not provided	Nigeria
	2009	KM822099	Humans	Not provided	Nigeria
	2009	KM822095	Humans	Not provided	Nigeria
	2009	KM822105	Humans	Not provided	Nigeria
	2009	KM822101	Humans	Not provided	Nigeria
	2009	KM822109	Humans	Not provided	Nigeria
	2009	KM822097	Humans	Not provided	Nigeria
	2009	KM822107	Humans	Not provided	Nigeria
	2011	JN651398	Humans	Serum	Edo State
	2011	JN651400	Humans	Serum	Adamawa State
	2011	JN651399	Humans	Serum	Ebonyi State
	2013	KJ944260	Humans	Serum	Ebonyi State
	2013	KJ944261	Humans	Serum	Nigeria
	2013	KJ944259	Humans	Serum	Nigeria
	Not provided	X52400	Not provided	Not provided	Nigeria

## 3 Results and Discussions

### 3.1 Existence of LF in Nigeria

**Figure 1:**
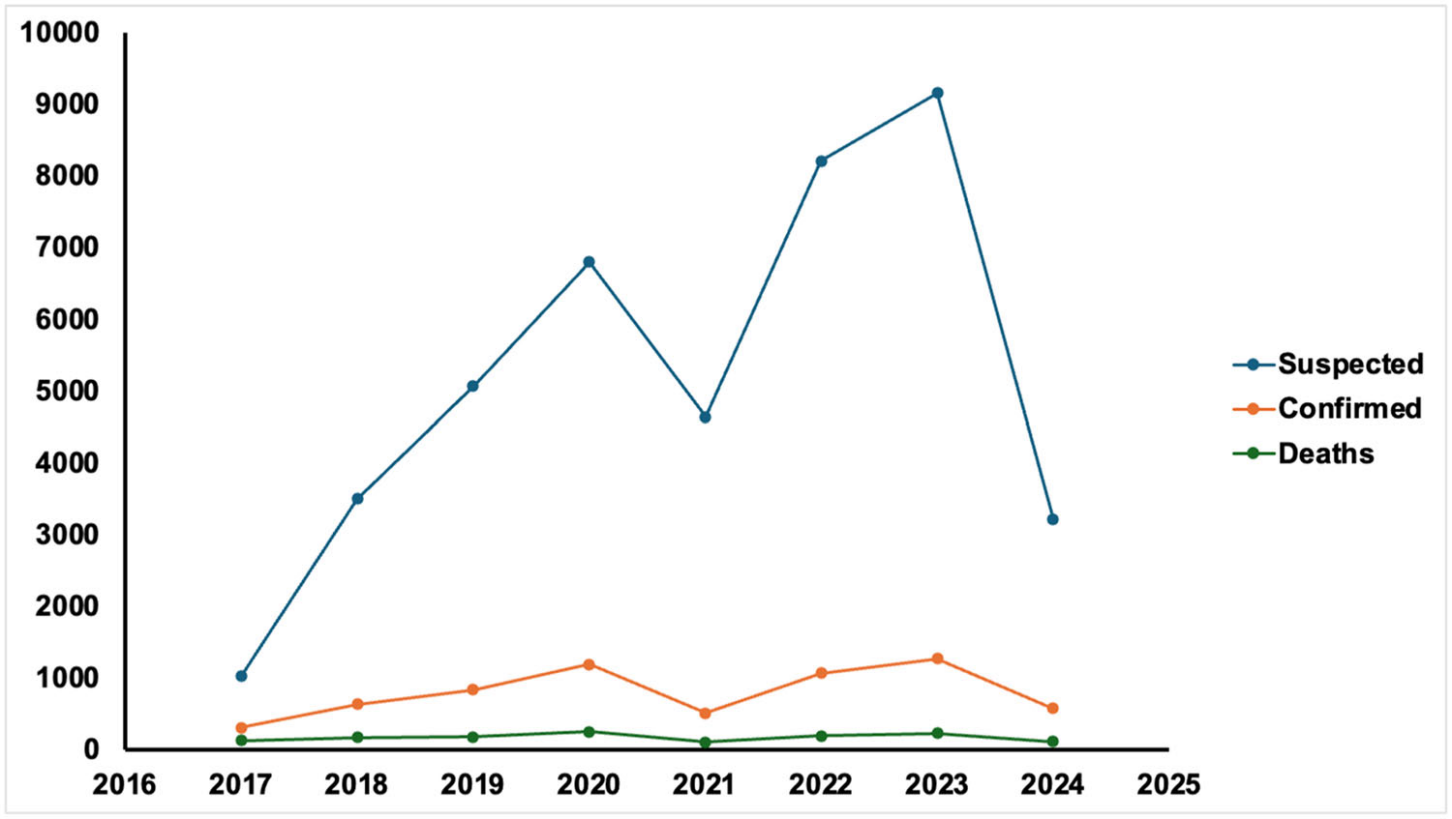
Trends of Lassa fever outbreak in Nigeria.

Nigeria has been shown to have the highest prevalence of LF among African countries (Figure 1) [23], with seasonal occurrence from November to April during the dry season and May to November during the wet season [24]. During this period, the farmers in the rural regions harvested their farm crops for storage, contributing to Nigeria's increasing rat population. Hence the foodstuffs in storage facilities such as silos and mud buildings are easily accessible to rodents. In addition, hunters are exposed to LF due to the encroachment of wild animals that are reservoirs of LFV during this period. Therefore, this may be the reason for the increase in LF during the dry season in Nigeria. Furthermore, during the dry season, there is an increase in migration, where individuals from the northern part of the country move to the southern region for business and other jobs. This may also increase the risk of transmission or spread of the virus. A study reveals a high magnitude of the disease in Nigeria, particularly in the country's southern region, with the high number of confirmed cases reported within the 6‐year study period also raising concerns [25]. The increased incidence of illnesses in the southern states may be caused by the presence of high numbers of reservoir hosts [26], cultural and environmental behaviors, such as food drying outdoors, or the presence of few significant treatment facilities in the states (where most of the nation's cases of LF are managed) [27]. A suspected case is considered an alert threshold; one confirmed case is an epidemic threshold. Therefore, LF is one of the seven epidemic‐prone diseases that must be reported in Nigeria under the Integrated Disease Surveillance System [28].

**Figure 2:**
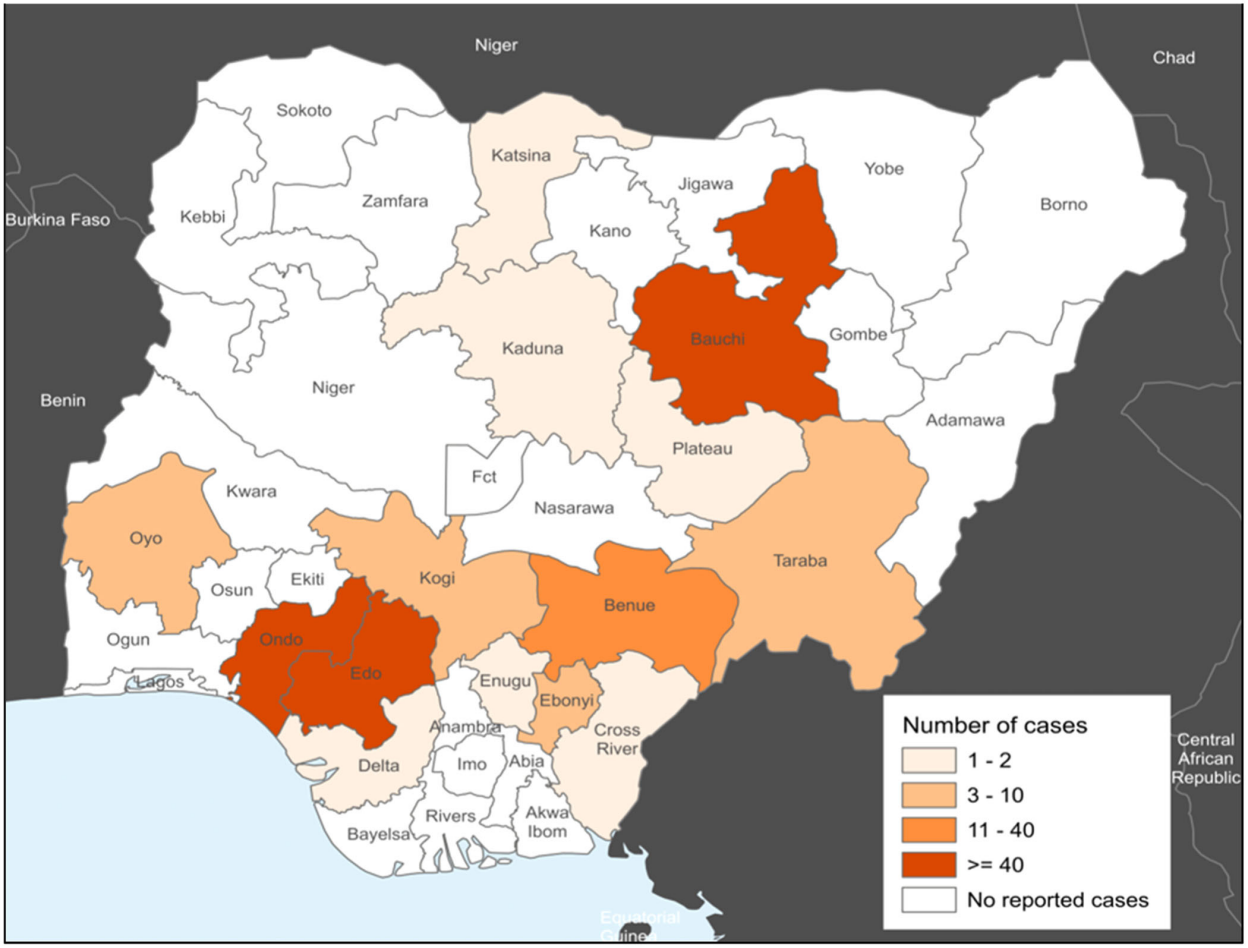
Map of Nigeria showing the affected states with Lassa fever (WHO).

Nigeria is comprised of 36 states organized into six geopolitical zones. Many states in the six geopolitical zones and the federal capital territory have experienced LF outbreaks (Figure 2) [29]. A study based on epidemiologic analysis of the temporal and spatial trends of the LF outbreak in Nigeria from 1969 to 2020 reveals that the country has been plagued by recurrent LF outbreaks, which have increased from irregular to regular annual and from a small localized outbreak to a nationwide outbreak [30]. In 2018, the nation faced its largest‐ever LF outbreak, which prompted the NCDC to activate the Emergency Operations Centre to manage the outbreak response [3].

**Figure 3:**
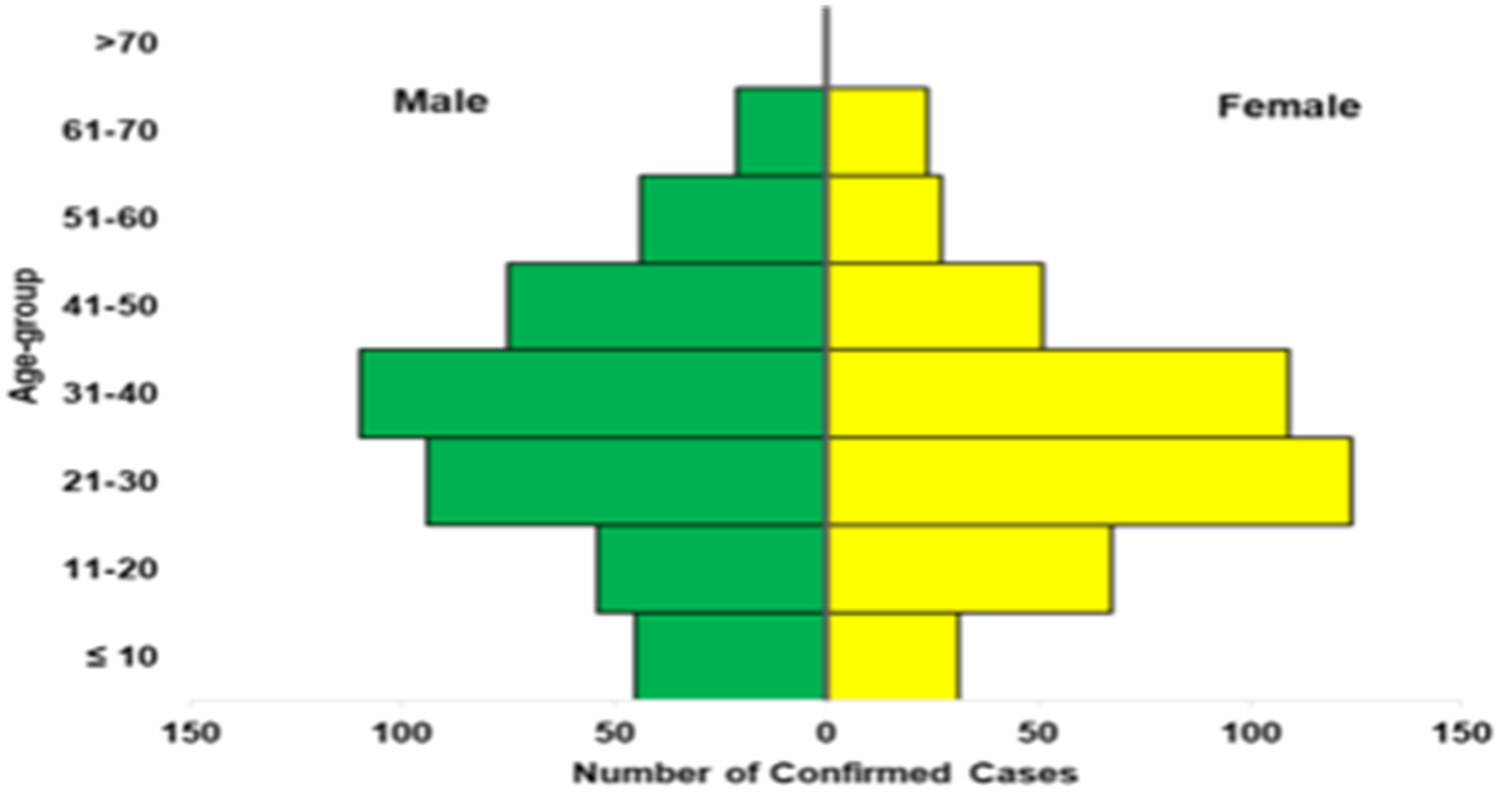
Relationship between 2024 reported Lassa fever cases and age group and sex in Nigeria [3].

In 2024, epidemiological data from NCDC confirmed that the predominant age group affected by LF is 31–40 years, and the male‐to‐female ratio for 2024 confirmed cases is 1:1 (Figure 3). However, a study by Ilori et al. [31] found that males were more susceptible to LF than women, but the reason is still unclear [31]. However, this may be due to activities done by men, such as hunting activities and the consumption of rodents. In addition, males migrate more frequently than females, and therefore, they are more exposed to the virus. Rodents are the primary viral reservoir host and frequent agricultural pests, and they are believed to be the direct cause of a vast majority of recorded LF cases. However, additional instances have emerged from hospital‐acquired illnesses and maybe from other small human‐to‐human transmission clusters [32].

### 3.2 Increase Occurrence of LF in Nigeria: What We Need to Know?

It was forecasted that zoonotic infections would increase because of an increase in temperature in sub‐Saharan Africa, making it a suitable factor for reservoir population growth [32]. In Nigeria, several factors may contribute to the rise and frequent occurrence of LF cases and mortality. Some of these factors come from infectious agents, reservoirs, the environment, and the socioeconomic condition of the country. Below, we discuss some vital points contributing to Nigeria's increased LF cases.

#### 3.2.1 Phylogenetic Analysis of LFV in Nigeria

**Figure 4:**
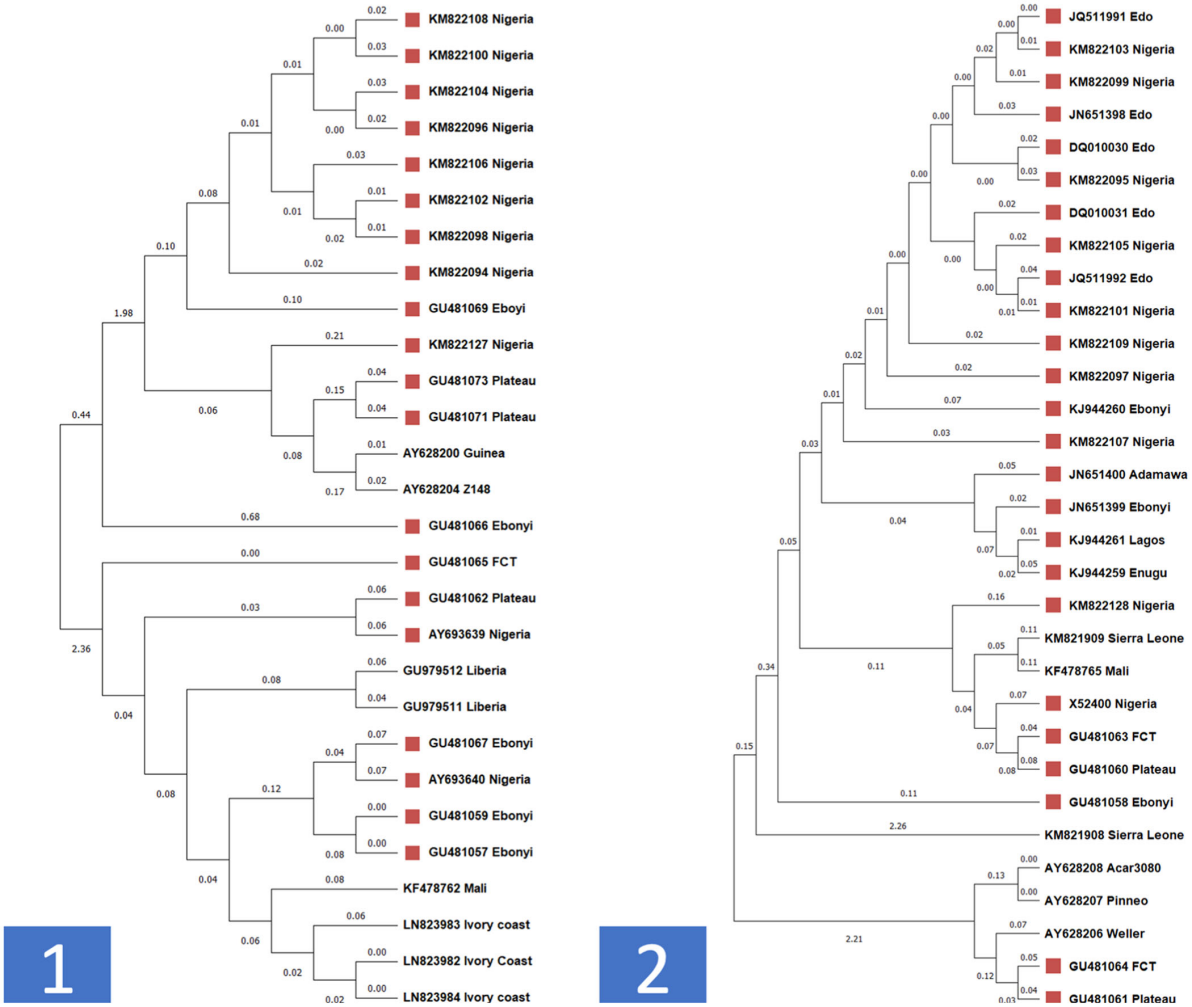
Phylogenetic tree of Lassa fever virus (1: Large segment; 2: Small segment) isolated from Nigeria and other African countries.

The phylogenetic tree of the virus showed a great relationship with several outbreaks of LF in Nigeria and other African countries. However, more data must be available on the molecular signature of the LFV in the states where outbreaks occur in Nigeria. This calls for more robust molecular diagnostic tools in Nigeria for better diagnosis. In the two segments of the LFV analyzed, the S segment is highly studied compared to segment L because of the high percentage of available sequences deposited in the NCBI database. The phylogenetic tree for the L segment (Figure 4 (1)) showed a closed clustering of Nigeria LFV isolates with other isolates from Guinea and LFV Z 148 strain. This indicates that most of the LFV circulating in Nigeria has a close evolutionary origin from Guinea. Our study corroborated with Ehichioya et al. study [33]. Nigeria is a coastal country, and part of its borders are shared with Guinea, in which the transmission of LF might have occurred through maritime means between both countries. In the past few years, flood breakouts have been a significant public health issue in Nigeria, and the flood might have exacerbated the increase in LF cases. Water is one of the transmission routes of the LFV. Efficient control of floods in Nigeria will reduce the exposure of people and animals from Nigeria to LF from other maritime bordering countries like Guinea. In the S segment, only a few sequences from Nigeria clustered with sequences from Sierra Leone and Mali. This finding was related to the study from Leski et al. [34] that revealed the link between Nigeria and Sierra Leone LFV lineages. The majority of the sequences from Nigeria clustered together, showing the close relatedness of each isolate to the states in Nigeria (Figure 3 (2)). This showed the endemicity of this LF strain in Nigeria and the cross‐transmission of LF across the borders of states in Nigeria.

#### 3.2.2 Increased Population

Nigeria is experiencing an increase in her population, which increases competition for food, ultimately leading to food scarcity in the community [35]. In rural areas, people hunt rodents to put food on their tables, exposing them to zoonotic diseases such as LF.

#### 3.2.3 Conflict and Violence

In 2023, around 3.3 million people were living in internally diplaced persons (IDPs) camps because of conflict and violence happening in Nigeria. The IDPs are faced with many challenges, such as poor housing facilities, medical facilities, and sanitation. The poor housing facilities and sanitation cause rodents to cohabitate with people, contributing to the transmission of LF to humans. Because of poor medical care, cases of LF are not detected early until it fully manifests and spreads among people, causing alarming threats in the country.

#### 3.2.4 Urbanization

Deforestation and mining activities in Nigeria are causing rodents to migrate from bushes to nearby human settlements. The rodents can access food storage facilities in households and markets, where they contaminate food materials via their urine and feces, exposing humans to LFV.

## 4 Conclusion and Recommendations

LF is an emerging zoonotic disease affecting Nigeria's public health and socioeconomic conditions. Therefore, multisectoral, collaborative, and transdisciplinary efforts are needed to curb the increasing rate of LF cases in the country. The integration of robust surveillance systems in the lens of One Health should be the priority of the NCDC in controlling the spread of LF in Nigeria. Rodent control in the rural settlement is another significant measure in preventing and controlling LF spread in Nigeria. Improving the house structure in the rural community should be considered; all cracks and holes should be closed in houses to avoid rodent breeding; rodenticide is also essential in controlling the rodent population in human settings. Proper use of foodstuffs storage facilities will also help prevent rodent contamination. Other public health measures include controlling floods, training healthcare workers on how to manage LF cases, and sanitation. Training on the proper use of personal protective equipment among healthcare providers should be a routine procedure in hospitals. Vaccine research should be supported in Africa, and research centers should come together to develop a vaccine for candidate LF. To our knowledge, scientists in the Centre for Advance Medical Research and Training, Usmanu Danfodiyo University Sokoto, Nigeria, are doing great work to develop a Lassa fever vaccine. Individuals in rural areas should be educated on the mode of Lassa fever transmission, dangers associated with rodent handling, and ways to prevent Lassa fever.

## Author Contributions

R.O.A. conceptualized the idea. R.O.A., I.I., A.A.B., J.F.A., and J.N.D.S. did the original draft, review, and editing. All authors have read and approved the final version of the manuscript; J.F.A. had full access to all of the data in this study and took complete responsibility for the integrity of the data and the accuracy of the data analysis.

## Ethics Statement

The authors have nothing to report.

## Conflicts of Interest

The authors declare no conflicts of interest.

## Data Availability

The authors confirm that the data supporting the findings of this study are available within the article.

## References

[1] Centers for Disease Control and Prevention (CDC) . Lassa Fever, https://www.cdc.gov/vhf/lassa/index.html#:~:text=About%20100%2C000%20to%20300%2C000%20infections,annually%2C%20with%20about%205%2C000%20deaths.

[2] J. R. Kay and J. B. Deborah , “Lassa Fever: Epidemiology, Clinical Features, and Social Consequences,” BMJ (London) 327 (2003): 1271–1275. 10.1136/bmj.327.7426.1271 PMC286250 14644972

[3] Nigeria Centre for Disease Control Weekly Epidemiological Report. Lassa Fever, https://ncdc.gov.ng/diseases/sitreps/?cat=5&name=An%20update%20of%20Lassa%20fever%20outbreak%20in%20Nigeria.

[4] W. Cao , M. D. Henry , P. Borrow , et al., “Identification of α‐Dystroglycan as a Receptor for Lymphocytic Choriomeningitis Virus and Lassa Fever Virus,” Science 282, no. 5396 (1998): 2079–2081, 10.1126/science.282.5396.2079. 9851928

[5] D. Kolawole , H. Raji , and M. I. Okeke , “Phylogenetic and Mutational Analysis of Lassa Virus Strains Isolated in Nigeria: Proposal for an In Silico Study,” JMIR Research Protocols 10, no. 3 (2021): e23015, 10.2196/23015. 33769296 PMC8088840

[6] S. L. M. Whitmer , T. Strecker , D. Cadar , et al., “New Lineage of Lassa Virus, Togo, 2016,” Emerging Infectious Diseases 24, no. 3 (2018): 599–602, 10.3201/eid2403.171905. 29460758 PMC5823357

[7] L. T. Mazzola and C. Kelly‐Cirino , “Diagnostics for Lassa Fever Virus: A Genetically Diverse Pathogen Found in Low‐Resource Settings,” BMJ Global Health 4 (2019): e001116. 10.1136/bmjgh-2018-001116 PMC6407561 30899575

[8] V. B. Meyer‐Rochow , K. Megu , and J. Chakravorty , “Rats: If You Can't Beat Them Eat Them! (Tricks of the Trade Observed Among the Adi and Other North‐East Indian Tribals),” Journal of Ethnobiology and Ethnomedicine 11 (December 2015): 45. 26024664 10.1186/s13002-015-0034-2 PMC4457996

[9] R. A. Keenlyside , J. B. McCormick , P. A. Webb , E. Smith , L. Elliott , and K. M. Johnson , “Case‐Control Study of *Mastomys Natalensis* and Humans in Lassa Virus‐Infected Households in Sierra Leone,” American Journal of Tropical Medicine and Hygiene 32 (1983): 829–837. 6881432 10.4269/ajtmh.1983.32.829

[10] O. Ogbu , E. Ajuluchukwu , and C. J. Uneke , “Lassa Fever in West African Sub‐Region: An Overview,” Journal of Vector Borne Diseases 44, no. 1 (2007): 1–11. 17378212

[11] S. Kernéis , L. Koivogui , N. Magassouba , et al., “Prevalence and Risk Factors of Lassa Seropositivity in Inhabitants of the Forest Region of Guinea: A Cross‐Sectional Study,” PLoS Neglected Tropical Diseases 3 (2009): e548. 19924222 10.1371/journal.pntd.0000548 PMC2771900

[12] O. B. Oluwafemi and W. A. Oluwatosin , “Davidson H. Hamer. Lassa Fever: An Evolving Emergency in West Africa,” American Journal of Tropical Medicine and Hygiene 104, no. 2 (2021): 466–473. 10.4269/ajtmh.20-0487 PMC7866331 33236712

[13] A. K. McElroy , R. S. Akondy , J. R. Harmon , et al., “A Case of Human Lassa Virus Infection With Robust Acute T‐Cell Activation and Long‐Term Virus‐Specific T‐Cell Responses,” Journal of Infectious Diseases 215 (2017): 1862–1872. 28863472 10.1093/infdis/jix201 PMC5853890

[14] Centers for Disease Control and Prevention . Lassa Fever. 2019, https://www.cdc.gov/vhf/lassa/index.html.

[15] S. Günther and O. Lenz , “Lassa Fever,” BMJ (London) 4 (1972): 253–254.

[16] S. H. Khan , A. Goba , M. Chu , et al., “New Opportunities for Field Research on the Pathogenesis and Treatment of Lassa Fever,” Antiviral Research 78 (2008): 103–115. 18241935 10.1016/j.antiviral.2007.11.003

[17] D. H. Walker , J. B. McCormick , K. M. Johnson , et al., “Pathologic and Virologic Study of Fatal Lassa Fever in Man,” American Journal of Pathology 107 (1982): 349–356. 7081389 PMC1916239

[18] A. N. Happi , C. T. Happi , and R. J. Schoepp , “Lassa Fever Diagnostics: Past, Present, and Future,” Current Opinion in Virology 37 (2019): 132–138. 31518896 10.1016/j.coviro.2019.08.002 PMC6768724

[19] D. U. Ehichioya , M. Hass , S. Ölschläger , et al., “Lassa Fever, Nigeria, 2005–2008,” Emerging Infectious Diseases 16, no. 6 (2010): 1040–1041. 20507773 10.3201/eid1606.100080 PMC3086228

[20] S. O. Babalola , J. A. Babatunde , O. M. Remilekun , et al., “Lassa Virus RNA Detection From Suspected Cases in Nigeria, 2011‐2017,” Pan African Medical Journal 34 (2019): 76. 31819792 10.11604/pamj.2019.34.76.16425 PMC6884721

[21] S. Kumar , G. Stecher , and K. Tamura , “MEGA 7: Molecular Evolutionary Genetics Analysis Version 7.0 for Bigger Datasets,” Molecular Biology and Evolution 33 (2016): 1870–1874. 27004904 10.1093/molbev/msw054 PMC8210823

[22] K. Tamura , “Estimation of the Number of Nucleotide Substitutions When There Are Strong Transition–Transversion and G+C‐content Biases,” Molecular Biology and Evolution 9 (1992): 678–687. 1630306 10.1093/oxfordjournals.molbev.a040752

[23] J. U. A. Grace , I. J. Egoh , and N. Udensi , “Epidemiological Trends of Lassa Fever in Nigeria From 2015‐2021: A Review,” Therapeutic Advances in Infectious Disease 8 (2021): 1–7. 10.1177/20499361211058252 PMC8637796 34868582

[24] K. C. Mofolorunsho , “Outbreak of Lassa Fever in Nigeria: Measures for Prevention and Control,” Pan African Medical Journal 23 (2016): 210. 27347299 10.11604/pamj.2016.23.210.8923 PMC4907747

[25] O. A. Okoro , E. Bamgboye , C. Dan‐Nwafor , et al., “Descriptive Epidemiology of Lassa Fever in Nigeria, 2012‐2017,” Pan African Medical Journal 37 (2020): 15. 10.11604/pamj.2020.37.15.21160 PMC7532845 33062117

[26] D. Agbonlahor , A. Erah , I. Agba , et al., “Prevalence of Lassa Virus Among Rodents Trapped in Three South‐South States of Nigeria,” Journal of Vector Borne Diseases 54, no. 2 (2017): 146–150. 28748835

[27] E. A. Ilori , C. Frank , C. C. Dan‐Nwafor , et al., “Increase in Lassa Fever Cases in Nigeria, January‐March 2018,” Emerging Infectious Diseases 25, no. 5 (2019): 1026–1027. 30807268 10.3201/eid2505.181247 PMC6478197

[28] N. A. Ajayi , C. G. Nwigwe , B. N. Azuogu , et al., “Containing a Lassa Fever Epidemic in a Resource‐Limited Setting: Outbreak Description and Lessons Learned From Abakaliki, Nigeria (January–March 2012),” International Journal of Infectious Diseases 17, no. 11 (2013): e1011–e1016. 23871405 10.1016/j.ijid.2013.05.015

[29] Nigeria Centre for Disease Control . An Update of Lassa Fever Outbreak in Nigeria Update 3.

[30] D. E. Agbonlahor , G. O. Akpede , C. T. Happi , and O. Tomori , “52 Years of Lassa Fever Outbreaks in Nigeria, 1969–2020: An Epidemiologic Analysis of the Temporal and Spatial Trends,” American Journal of Tropical Medicine and Hygiene 105, no. 4 (2021): 974–985. 34460421 10.4269/ajtmh.20-1160 PMC8592130

[31] E. A. Ilori , Y. Furuse , O. B. Ipadeola , et al., “Epidemiologic and Clinical Features of Lassa Fever Outbreak in Nigeria, January 1–May 6, 2018,” Emerging Infectious Diseases 25, no. 6 (2019): 1066–1074. 31107222 10.3201/eid2506.181035 PMC6537738

[32] New Nigerian Lassa Fever Outbreak Underway–and Expanding its Range, https://healthpolicy-watch.news/new-nigerian-lassa-fever-outbreak/.

[33] D. U. Ehichioya , M. Hass , B. Becker‐Ziaja , et al., “Current Molecular Epidemiology of Lassa Virus in Nigeria,” Journal of Clinical Microbiology 49, no. 3 (2011): 1157–1161. 21191050 10.1128/JCM.01891-10 PMC3067713

[34] T. A. Leski , M. G. Stockelman , L. M. Moses , et al., “Sequence Variability and Geographic Distribution of Lassa Virus, Sierra Leone,” Emerging Infectious Diseases 21, no. 4 (2015): 609–618. 25811712 10.3201/eid2104.141469 PMC4378485

[35] R. O. Adesola , E. Opuni , I. Idris , et al., “Navigating Nigeria's Health Landscape: Population Growth and Its Health Implications,” Environmental Health Insights 18 (2024): 11786302241250211. 38698838 10.1177/11786302241250211 PMC11064746

